# The critical role of histology in distinguishing sarcoidosis from common variable immunodeficiency disorder (CVID) in a patient with hypogammaglobulinemia

**DOI:** 10.1186/s13223-019-0383-9

**Published:** 2019-12-02

**Authors:** Rohan Ameratunga, Yeri Ahn, Dominic Tse, See-Tarn Woon, Jennifer Pereira, Sinead McCarthy, Hilary Blacklock

**Affiliations:** 1 Department of Virology and Immunology, Auckland City Hospital, Park Rd, Grafton, Auckland, 1010 New Zealand; 2 Department of Neurology, Auckland City Hospital, Park Rd, Grafton, Auckland, 1010 New Zealand; 3 Department of Histopathology, Auckland City Hospital, Park Rd, Grafton, Auckland, 1010 New Zealand; 4 Department of Molecular Medicine and Pathology, Faculty of Medical and Health Sciences, University of Auckland, Auckland, New Zealand; 5 Department of Haematology, Middlemore Hospital, Auckland, New Zealand

**Keywords:** CVID, Sarcoidosis, IVIG, Neurosarcoidosis, Diagnostic criteria

## Abstract

**Background:**

Common variable immunodeficiency disorders (CVID) are a rare group of primary immune defects, where the underlying cause is unknown. Approximately 10–20% of patients with typical CVID have a granulomatous variant, which has closely overlapping features with sarcoidosis.

**Case presentation:**

Here we describe a young man who sequentially developed refractory Evans syndrome, cauda equina syndrome and most recently renal impairment. Following immunosuppression, he has made a recovery from all three life-threatening autoimmune disorders. As the patient was hypogammaglobulinemic for most of the time while on immunosuppression, vaccine challenges and other tests were not possible. Histological features were in keeping with sarcoidosis rather than the granulomatous variant of CVID. In the brief period when immunosuppression was lifted between the cauda equina syndrome and renal impairment, he normalised his immunoglobulins, confirming sarcoidosis rather than CVID was the underlying cause.

**Conclusion:**

We discuss diagnostic difficulties distinguishing the two conditions, and the value of histological features in our diagnostic criteria for CVID in identifying sarcoidosis, while the patient was hypogammaglobulinemic. The key message from this case report is that the characteristic histological features of CVID can be very helpful in making (or excluding) the diagnosis, particularly when other tests are not possible.

## Background

Common variable immunodeficiency disorders (CVID) are a rare group of primary immunodeficiency disorders (PIDs) leading to immune system failure (ISF) caused by late onset antibody failure (LOAF). The majority of CVID patients have profound hypogammaglobulinemia associated with antibody defects [1]. The genetic basis is unknown in the majority of patients with a CVID phenotype. If a causative mutation is identified, patients are removed from the umbrella diagnosis of CVID and are deemed to have a CVID-like disorder consequent to a specific PID [2].

Most patients with CVID and CVID-like disorders are predisposed to recurrent infections because of ISF. Approximately 10–15% of patients with typical CVID have clinical features which closely resembles sarcoidosis [3–6]. This has been termed the granulomatous variant of CVID (GVCVID). Patients with GVCVID appear to have an increased risk of autoimmunity [7].

GVCVID and sarcoidosis can be very difficult to distinguish because of closely overlapping clinical features (Table 1) [7]. As with sarcoidosis, GVCVID typically involves the lung, liver and lymph nodes. A variety of thoracic radiological features can be seen including lymphadenopathy and interstitial lung disease [8, 9]. The pulmonary changes are collectively referred to as granulomatous interstitial lung disease (GLILD) [7, 10]. Histological correlates of GLILD include non-caseating granulomas, follicular bronchiolitis and lymphoid interstitial pneumonitis. In contrast to sarcoidosis, plasma cells are absent in GVCID and germinal centres are often poorly formed or disrupted.

**Table 1:** Differential diagnosis: comparison of the features of GVCVID with sarcoidosis

Parameter	GVCVID	Sarcoidosis	Comment
Clinical features			
Infections	More common	Uncommon	Favours sarcoidosis
Lymphadenopathy	Common	Common	Does not differentiate
Evans syndrome	Relatively common	Exceedingly rare	Only two cases described in sarcoid: strongly favours CVID
Interstitial lung disease	Less common	Common	No obvious interstitial lung disease: favours CVID
Steroid responsive renal disease	Very rare	Described in sarcoidosis	Strongly favours sarcoidosis
Raised intracranial pressure	Rare	More common	Favours sarcoidosis
MRI showing cauda equina involvement	No reports	Very rare	Cauda equina involvement described only in sarcoidosis
Laboratory features			
Switched memory B cells absent	Consistent with CVID	Reduced memory B cells	Favours CVID
Angiotensin converting enzyme levels (ACE)	ACE levels can be elevated in GVCVID	ACE levels can be normal in sarcoidosis	Non discriminatory
Absent TRECs	Favours LOCID but on MMF	Not described	Favours CVID
CSF findings: only increased protein		Cells expected	Favours CVID
Initial IgG normal but subsequent decrease, but normalised after stopping immunosuppression	Decreased	Increased	Strongly favours sarcoidosis and excludes CVID
Lymph node: disrupted architecture	Disrupted architecture in CVID	Plasma cells and germinal centres present	Strongly favours sarcoidosis

Most clinical and laboratory abnormalities can occur in both disorders. Overall the findings strongly favour sarcoidosis. The normalisation of IgG and the histological findings subsequently excluded CVID. See text for abbreviations

There is no single clinical or laboratory feature which is pathognomonic for CVID. In areas of uncertainty diagnostic criteria can be very useful. The ESID/PAGID (1999) and ICON (2016) diagnostic criteria for CVID emphasise poor vaccine responses [11, 12]. Serological responses to vaccines can however be difficult to assess when patients are either being treated with subcutaneous or intravenous immunoglobulin (SCIG/IVIG) or when immunosuppressed for autoimmunity. These vaccine based CVID diagnostic criteria are thus difficult to apply in complex cases such as the patient described here. Furthermore, our recent prospective NZ Hypogammaglobulinemia study (NZHS) has shown the unreliability of vaccine responses in distinguishing patients with symptomatic and asymptomatic hypogammaglobulinemia, who have an excellent long-term prognosis [13]. The ESID/PAGID (1999) and ICON (2016) criteria do not include any of the characteristic histological features of CVID.

In 2013, we described new diagnostic criteria for CVID [14, 15]. These criteria include clinical, serological and histological features of the disorder, which allows a more precise diagnosis. CVID was previously a diagnosis of exclusion, but can now be made with greater precision. To fulfil our criteria, patients are required to have significant symptomatic hypogammaglobulinemia with no other explanation for the disorder. Supportive serological markers including poor or transient vaccine responses, absent isohemagglutinins, IgG3, IgA or IgM deficiency along with tests for significant autoimmunity. Importantly, our CVID diagnostic criteria also include histological features of the disorder including absence of plasma cells. Our reasoning was that histological features may be useful, when SCIG/IVIG treatment or immunosuppression preclude assessing serological tests. They can also be helpful in historical cases where the patient is deceased, if there are histological specimens from previous investigations. While these histological features can occur in other disorders, the primary symptomatic hypogammaglobulinemia confers specificity for CVID in our criteria.

Here we describe the value of histology in excluding CVID in a patient who was immunosuppressed for life-threatening autoimmunity and placed on IVIG for severe hypogammaglobulinemia. The histological features were strongly indicative of sarcoidosis rather than GVCVID. The patients IgG subsequently normalised when immunosuppression was briefly lifted, confirming that sarcoidosis rather than GVCVID was the underlying disorder. A normal IgG excludes CVID in all current diagnostic criteria.

## Case presentation

The 22-year-old male patient presented at age 14 years with epistaxis related to severe thrombocytopenia. Immune thrombocytopenic purpura (ITP) was diagnosed and he was initially treated with high doses of prednisone. Prior to immunosuppression, his immunoglobulins had been measured and were found to be in the normal range (IgG 8.8 g/l, nr 7–14). Following initiation of steroid therapy he became hypogammaglobulinemic (IgG 4.3 g/l).

Once the severe hypogammaglobulinemia was identified, he was treated with IVIG (2 g/kg), with the added expectation the immunomodulatory doses of IVIG would reduce his risk of infections as well as potentially benefitting his ITP. He then developed Evans syndrome with autoimmune haemolytic anaemia, in addition to the ITP. When reviewed in 2011, he was Cushingoid from corticosteroids. He responded to this regimen and the prednisone was tapered to 10 mg daily and stopped.

He was well until 2013 when his ITP relapsed. On this occasion he did not respond to high dose prednisone, vincristine, cyclosporine, cyclophosphamide or rituximab. Due to ongoing cytopenias he underwent a splenectomy in 2014. Histology revealed the presence of splenic granulomas. Although the splenectomy was not successful, he remitted with eltrombopag in 2014.

He remained well until early 2017 when he developed diplopia consequent to bilateral cranial VI nerve palsies. He had headaches, fever and signs of meningism. Lumbar puncture showed an elevated CSF opening pressure but protein and other cellular parameters were normal. CSF bacterial culture, fungal antigen tests and viral PCR studies excluded infection. MRI of the brain showed an ill-defined enhancing area around the anterior pons.

**Fig. 1:**
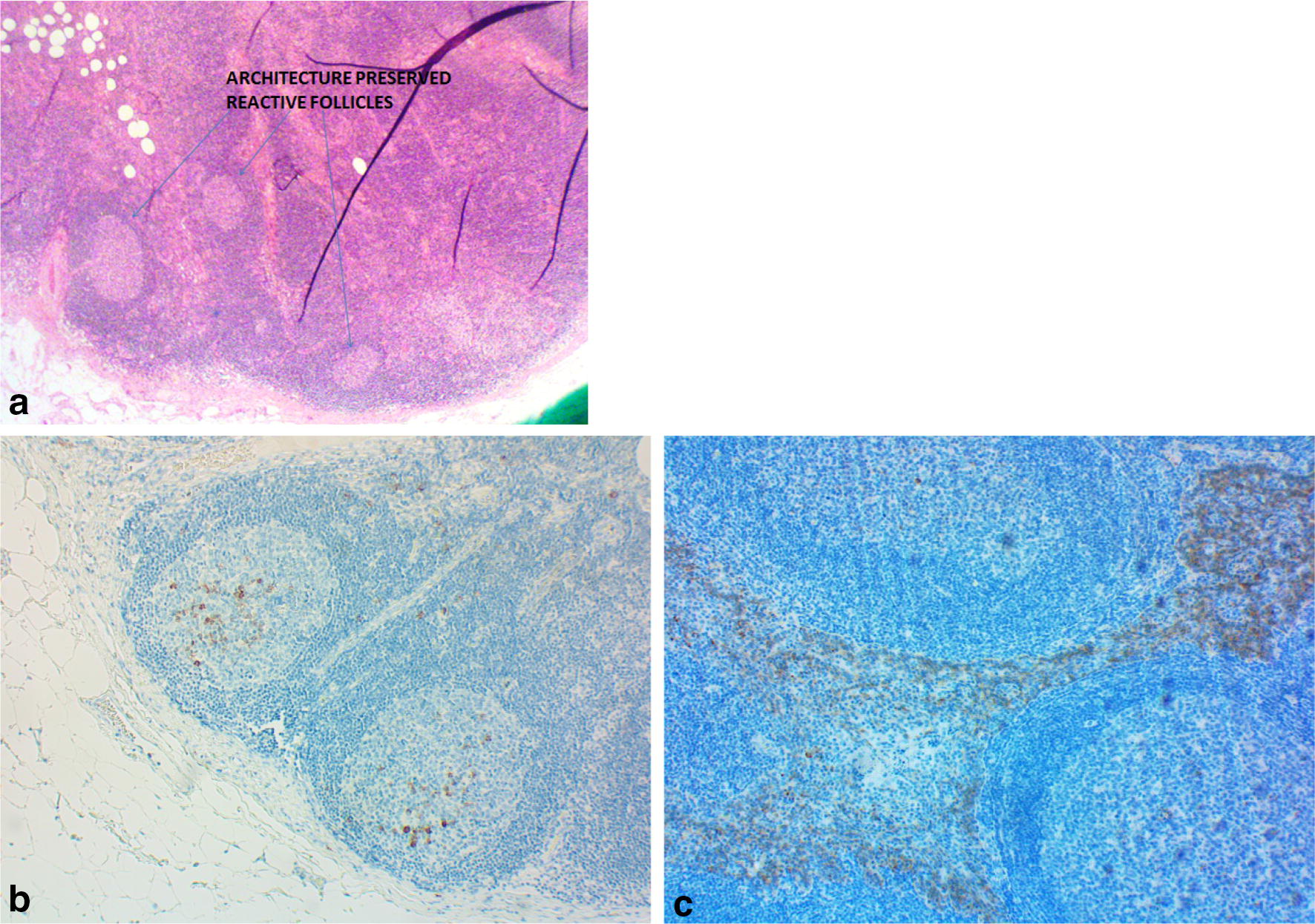
Lymph nodes from the patient (**a**, **b**) and normal control (**c**). Plasma cells staining with CD138 are shown in brown. **a** The patient has normal lymph node architecture. **b** Showing reduced numbers of plasma cells in the germinal centre in the patient. **c** The plasma cells are in the interfollicular areas in the control. CVID is typically associated with the absence of plasma cells and poorly formed germinal centres. The patient was not on immunosuppression at the time of the lymph node excision

He had widespread lymphadenopathy including mediastinal and abdominal lymphadenopathy on MRI scanning. Excision biopsy of a left inguinal lymph node showed the presence of discrete non-caseating granulomas, reactive follicles and overall normal lymph node architecture (Fig. 1a). Plasma cells were present although slightly reduced compared to a normal control (Fig. 1b, c). His serum angiotensin converting enzyme (ACE) level was normal.

**Fig. 2:**
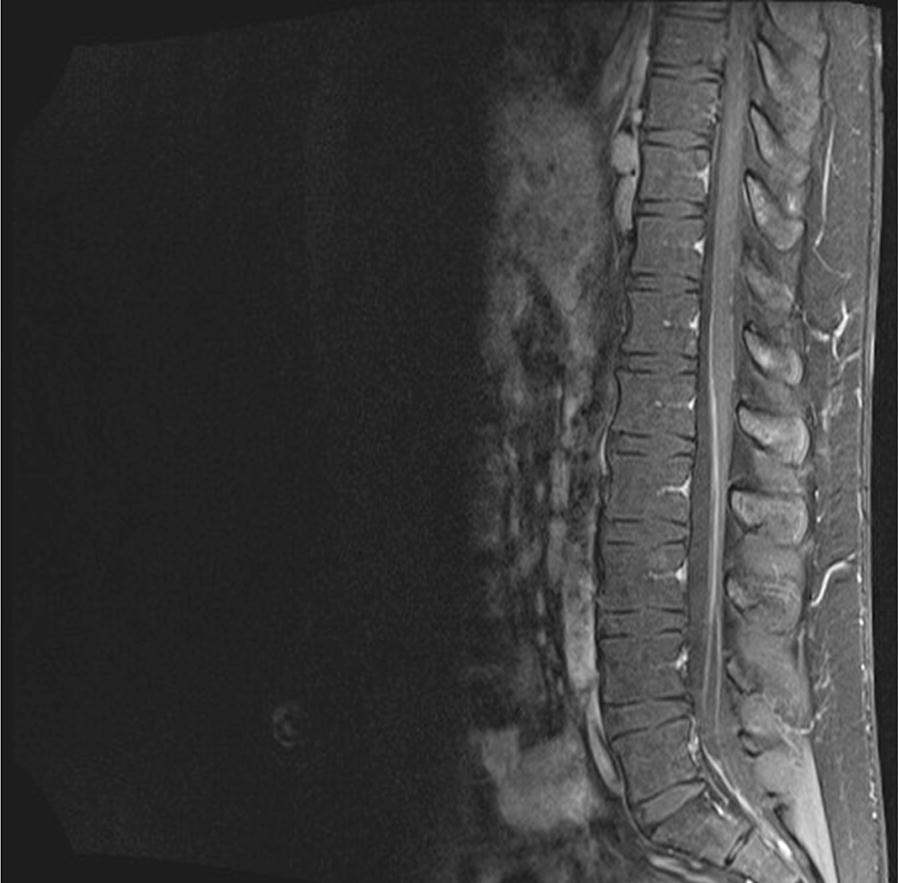
MRI of the cauda equina. Enhancing lesions of the nerve roots are seen consistent with granulomatous inflammation. A biopsy was not possible, given the location

The following week he developed increasing gait instability and was readmitted to hospital. Repeat CSF examination again showed an increased opening pressure (23 cm) and a raised protein level. The cell count and glucose were again normal. A repeat MRI scan of brain and spine revealed enhancing lesions in the cauda equina roots suggestive of granulomatous inflammation (Fig. 2). The previous pontine lesion had resolved. Given the location of inflammation, a biopsy was not possible. Nerve conduction tests of the lower limbs were consistent with nerve root involvement. Several months after starting steroids, a gallium PET scan showed enhancing lesions of the lacrimal gland.

PCR studies of the CSF and blood were negative for toxoplasma and tuberculosis (TB), the two main infectious differential diagnoses for granulomatous infections in New Zealand. He had not recently travelled internationally. He had a good response to IV methylprednisolone (1 g) treatment with resolution of headaches and fever, as well as improvement in gait. He was able walk unaided. The improvement was reflected in the blood tests, with the CRP decreasing from 69 to 7 mg/l within a week, and improving cytopenias. He was placed on low dose oral prednisone and mycophenolate with iv methylprednisolone 1 g every 2 weeks.

**Fig. 3:**
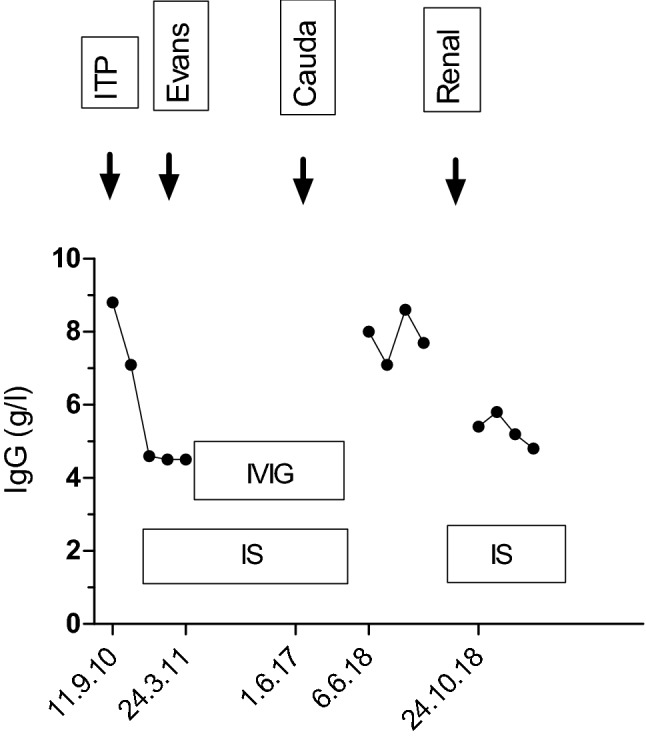
IgG levels during immunosuppression. In mid-2018 the IgG normalised briefly between resolution of the cauda equine syndrome and treatment of renal disease. *Cauda* Cauda equine syndrome, *Evans* Evans syndrome, *ITP* immune thrombocytopenia, *IVIG* intravenous immunoglobulin, *IS* immunosuppression, *Renal* renal impairment

In mid-2018 when the immunosuppression was lifted, his IgG increased from 3.0 g/l to 8.8 g/l (Fig. 3) and the previously decreased IgA and IgM also normalised. Shortly afterwards he developed progressive renal impairment with a creatinine clearance of 36 ml/min. Renal biopsy showed 18/42 sclerosed glomeruli. No granulomas were identified. He was recommenced on Mycophenolate (MMF) and low dose prednisone. There was insufficient time to undertake vaccine response or detailed immunophenotyping studies before the immunosuppression was urgently recommenced. Over the following 6 months the creatinine clearance increased to 66 ml/min. The family has undergone whole exome sequencing for diagnosis and gene discovery but no causative mutation was identified [16].

## Discussion and conclusion

This patient illustrates the difficulty in distinguishing sarcoidosis from the granulomatous variant of CVID because of the closely overlapping features (Table 1) [3]. Both are associated with organ dysfunction caused by multisystem granulomas [17]. There is a marked difference in long-term prognosis between the two conditions [18]. There are other important therapeutic implications in determining the exact underlying condition. Patients without an underlying immunodeficiency may be better able to tolerate immunosuppression. Some drugs such as TNF inhibitors may be more effective for neurosarcoidosis than GVCVID.

Approximately twenty-five percent of CVID patients have a causative mutation and if identified are classified as having a CVID-like disorder [19, 20]. All current CVID diagnostic criteria exclude patients with a known disorder, including causative mutations. This is the basis of separating CVID from CVID-like disorders. Identification of an underlying genetic defect also has profound implications for the family [19–21]. In contrast, no underlying causative genetic defect has been identified in sarcoidosis. We did not identify a causative mutation in this family. The absence of a mutation does not exclude either CVID or sarcoidosis.

Sarcoidosis and GVCVID are associated with lymphadenopathy and the lungs are involved in both disorders, although there may be subtle differences in the radiological findings between these two disorders [7]. Compared to GVCVID the majority of patients with sarcoidosis have interstitial lung disease [7]. The absence of interstitial lung disease in our patient favours GVCVID [8].

The neurological disease is more in keeping with sarcoidosis [22]. Raised intracranial pressure (ICP) has been described in neurosarcoidosis and may have been the explanation for the headaches and the bilateral VI cranial nerve palsies [23]. The raised ICP may have been either from previous prednisone treatment and/or meningeal involvement of the granulomas [24]. Although intracranial disease has been identified in GVCVID [25], cauda equina involvement has not. In one CVID patient with cauda equina syndrome, the authors felt their patient with CVID had concomitant neurosarcoidosis [26]. We therefore consider that our patient’s cauda equina syndrome to be more consistent with sarcoidosis.

Our patient had normal ACE levels. ACE levels can be elevated or normal in both GVCVID and sarcoidosis and are therefore non-discriminatory [27, 28]. Our patient had reduced memory B cells and absent switched memory B cells. We have shown variability in B cell numbers over time, repeat tests (4 years following last rituximab dose) but again showed absence of switched memory B cells [29]. Although reduction in switched memory B cells is also seen in sarcoidosis [30], complete absence would favour CVID. He was however on immunosuppression at the time.

Autoimmune cytopenias, are very rare in sarcoidosis [7]. ITP [31] and AIHA [32–34] have been individually been described in sarcoidosis but the combination leading to Evans syndrome appears to be exceptionally rare [35, 36]. In contrast, uveitis and autoimmune/inflammatory skin disease is more common in sarcoidosis [24]. The Evans syndrome in our patient therefore strongly favours CVID.

Although controversial, [37] vaccine challenge responses might be expected to be impaired in CVID compared with sarcoidosis but were not possible because the patient was already receiving intravenous immunoglobulin or immunosuppression for most of the last decade [38]. We have previously discussed the difficulties with the interpretation of vaccine responses in CVID [11]. We have recently shown the poor utility of vaccine responses in patients with primary hypogammaglobulinemia [13]. Even if we had been able to undertake vaccine challenge responses, they may not have excluded CVID.

The patient had absent T cell receptor excision circles (TRECS) suggestive of a severe T cell defect, although these were undertaken while on mycophenolate and oral prednisone. He has not suffered severe viral or opportunistic infections. There was no clinical suspicion of late onset combined immunodeficiency (LOCID), with viral and opportunistic infections [39]. Similarly, we did not undertake extended immunophenotyping because of the immunosuppression, given the potential difficulties interpreting the results.

Overall, the histological findings were strongly in favour of sarcoidosis (Table 1) [40]. Careful review of his lymph node histology and immunohistochemistry studies showed the presence of CD138 + staining plasma cells (Fig. 1b) and intact germinal centres. Although plasma cells were reduced compared to the control (Fig. 1c), their presence is diagnostic of sarcoidosis, in the absence of other granulomatous disorders.

His initial IgG was in the normal range but rapidly decreased following immunosuppression. Typically patients with sarcoidosis have elevated IgG levels, while CVID patients have levels < 5 g/l [15]. Immunosuppressive therapy such as rituximab can unmask CVID [41]. His IgG increased into the normal range when his immunosuppression was decreased, excluding CVID [42].

Prior to recovery of his IgG, he had many of the features of the revised ESID registry (2014) criteria for CVID as he had a granulomatous disorder, reduction in IgG and IgA, absent switched memory B cells with an age of onset greater than 4 years. He did not however meet the revised ESID registry (2014) criteria because of the immunosuppression, where a secondary cause could not be excluded [43]. Absence of plasma cells are not part of the revised ESID registry (2014) criteria, which is probably the most important clue to this clinical conundrum, in allowing the correct diagnosis.

We have previously shown that histological features of our diagnostic criteria were similarly helpful in another patient who had both CVID and drug induced hypogammaglobulinemia (Category D in the Ameratunga et al. criteria) [44]. This case report highlights the value of histology in being able to make a firm diagnosis, where other tests were not possible or inconclusive. Given the difficulties interpreting vaccine challenge responses in patients with hypogammaglobulinemia, histology is likely to much more useful in the diagnosis of similar patients.

## Data Availability

No additional data is available for this study.
